# Imported Talaromycosis in Oman in Advanced HIV: A Diagnostic Challenge Outside the Endemic Areas

**DOI:** 10.1007/s11046-017-0124-x

**Published:** 2017-03-04

**Authors:** Jalila Mohsin, Sulin Al Khalili, A. H. G. Gerrits van den Ende, Faryal Khamis, Eskild Petersen, G. Sybren de Hoog, Jacques F. Meis, Abdullah M. S. Al-Hatmi

**Affiliations:** 1 Department of Medical Microbiology, Royal Hospital, Muscat, Oman; 2 Westerdijk Fungal Biodiversity Institute, PO Box 85167, 3508 AD Utrecht, The Netherlands; 3 Department of Infectious Diseases, The Royal Hospital, Muscat, Oman; 4 Institute of Clinical Medicine, Aarhus University, Aarhus, Denmark; 5 Directorate General of Health Services, Ministry of Health, Ibri, Oman; 6 Centre of Expertise in Mycology Radboudumc/Canisius Wilhelmina Hospital, Nijmegen, The Netherlands; 7 Department of Medical Microbiology and Infectious Diseases, Canisius-Wilhelmina Hospital, Nijmegen, The Netherlands

**Keywords:** *Talaromyces marneffei*, Talaromycosis, *Penicillium marneffei*, Penicilliosis, HIV, Travel-related infections, Oman

## Abstract

A 37-year-old male living in Oman was seen by his physician with complaints of cough, body aches with bilateral lower limb weakness and on and off fever. He was diagnosed with HIV infection and culture from blood and bone marrow grew *Talaromyces marneffei*. He had travelled to Malaysia on several occasions. Treatment with liposomal amphotericin B resulted in complete cure. This case is reported for its rarity and unusual presentation to alert clinicians and microbiologists to consider *T. marneffei* as an etiology in high risk individuals. Our case is the first recorded diagnosis of *T. marneffei* in Oman.

## Background

*Talaromyces marneffei* (formerly *Penicillium marneffei*) [1] is a facultative intracellular pathogen capable of causing disseminated infections in humans with wild bamboo rats in Southeast Asia as the natural reservoir [2, 3]. The first case of talaromycosis was reported in 1973 from an American minister who had Hodgkin’s disease and been living in Southeast Asia [4]. In 1984, the second reported case also concerned an American citizen, who had travelled in Thailand [5]. Four years later, *T. marneffei* was identified in HIV-infected individuals [6], and soon it emerged as an AIDS-defining disease in endemic regions [7]. Talaromycosis has become an important risk to immunocompromised travelers with impaired acquired immunity to these regions, particularly Vietnam, Laos, Malaysia, Myanmar, East India, Thailand and the Guang Xi province in southeastern China. Numerous cases of talaromycosis have been described in patients with a latently compromised immune system returning from these endemic areas [8], but also in organ transplant recipients from donors originating from these regions [9].

Patients usually present with fungal invasion of multiple organ systems such as blood, bone marrow, skin and lungs [10]. *Talaromyces marneffei* infection has a high mortality, when diagnosis and treatment are delayed. Mostly, cases of talaromycosis are seen in patients having CD4 T-lymphocyte cell counts <100 cells/mL [11]. Although the vast majority of patients with talaromycosis are HIV-infected, *T. marneffei* infection has been documented among patients with other types of impaired cell-mediated immunity [2].

With increasing international travel, *T. marneffei* is becoming a threat to tourism to the tropics. Imported cases of this mycosis to the Middle East have as yet not been reported. To the best of our knowledge, our case from the Middle East is the first example of disseminated *T. marneffei* infection in an HIV-diagnosed patient who visited an endemic area.

## Case Report

A 37-year-old male was admitted on the June 4, 2016, at a secondary care hospital in Oman with a history of anorexia, significant weight loss of 30 kg over a period of 5 weeks, generalized body pain with bilateral lower limb weakness and periodic fever. Blood cultures were initially reported negative. Preliminary investigations for pyrexia of unknown origin, and blood and urine cultures and serology for *Brucella* and Q-fever were negative. However, human immunodeficiency virus (HIV-1&2) testing by a fourth generation enzyme-linked immunosorbent assay (ELISA) turned out to be positive.

Four days post-admission, the patient developed a generalized, vesicular, non-umbilicated rash over face, arms, trunk front and back and both legs clinically suggestive of chickenpox, which was treated with intravenous acyclovir. Unfortunately, no samples were taken from the skin lesions or from blood to confirm this infection. In the third week of admission, he was transferred to the Royal Hospital, a tertiary health care facility for additional investigations and management. He had a history of traveling to Malaysia several times for reasons that were unrevealed; the latest was 2 months prior to his admission lasting 10 days. He denied any contact with animals including rats.

**Fig. 1:**
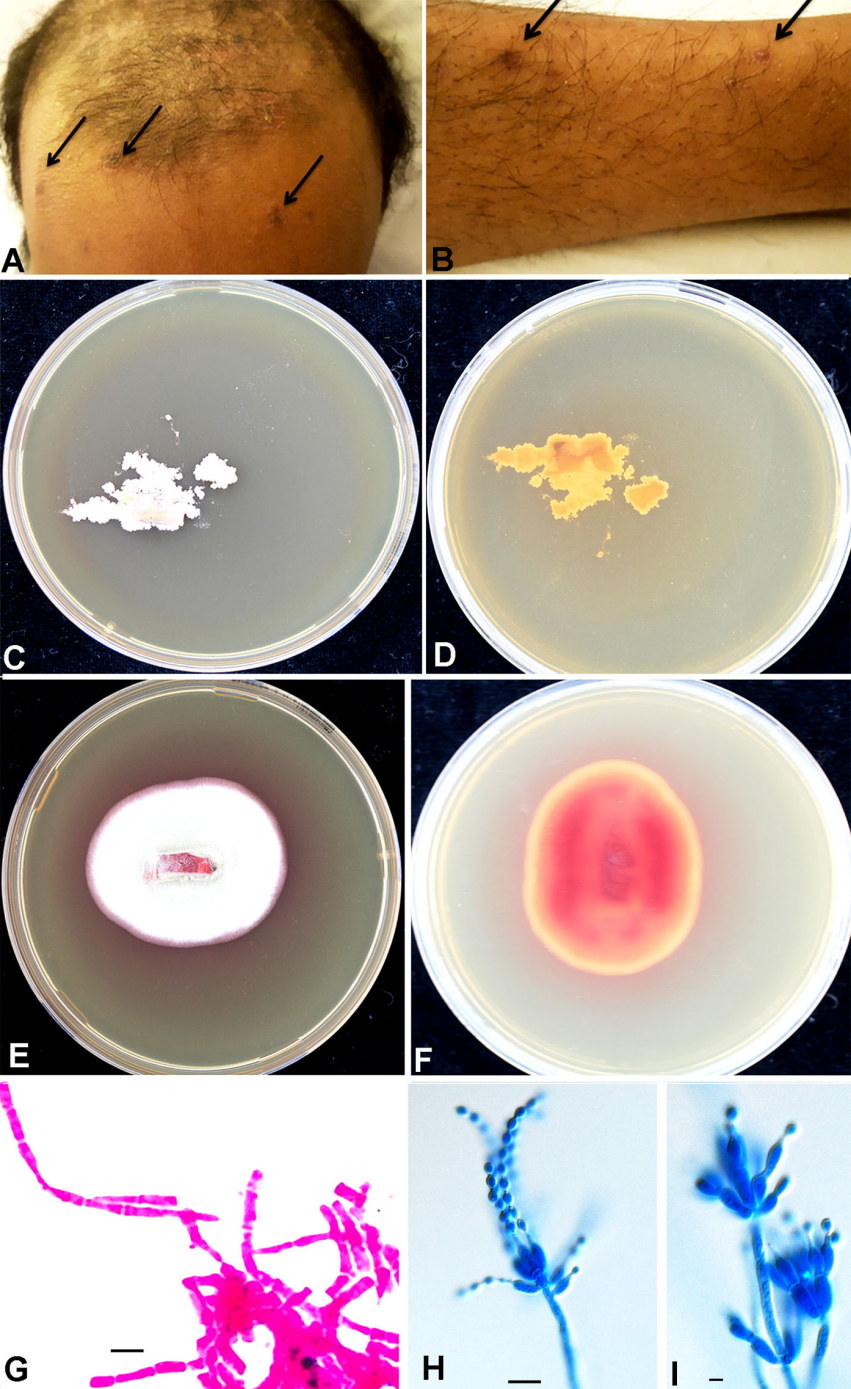
**a**, **b** Figs. 1 and 2: crusted dry skin lesions over forehead and left tibia. **c**, **d** Growth of the isolate *T. marneffei* on SAB agar, front white yeast-like growth and the reverse is *light brownish* after 3 d. **e**, **c** *White greenish* centered powdery fungal colonies with distinctive *red* diffusible pigment on Sabouraud’s dextrose agar, consistent with the characteristics of *T. marneffei*. **g** Gram stain of the initial fungal growth at 37 °C with arthroconidia. **h** , **i** Conidiophores. *Scale bar*: 10 μm. (Color figure online)

The patient complained of bilateral lower limb pain, numbness and weakness. He was fully awake and had preserved patellar and achilles reflexes and down-going planters (negative Babinski). He had crusted, dry, papulo-macular skin lesions in the face, head and extremities (Fig. 1a, b). He was not in distress and had no jaundice or palpable lymphadenopathy. Physical examination showed that the patients was emaciated, pale and depressed, with temperature of 38.1 °C. The Glasgow Coma Scale (GCS) was 15/15 without slurred speech but with weak wasted limbs. He also had oral candidiasis which is one of the indicators for a possible HIV infection. Computed Tomography (CT) of the brain was suggestive of minimal periventricular white matter hypodensity, crowding of the gyri with obliteration of the CSF spaces at the convexity of the brain, while a chest CT showed significant mediastinal and hilar adenopathy as well as a right lower lobe intra-parenchymal lymph node. An ill-defined, small ground-glass opacity was seen in the right middle lobe of lung. An abdominal CT showed splenomegaly with mild hepatomegaly. The radiological differentials included tuberculosis, lymphoma or reactive adenopathy related to HIV infection. A nerve conduction study showed an acute distance dependent axonal sensorimotor polyneuropathy consistent with chronic symmetrical polyneuropathy. The patient refused a lumbar puncture and MRI.

**Fig. 2:**
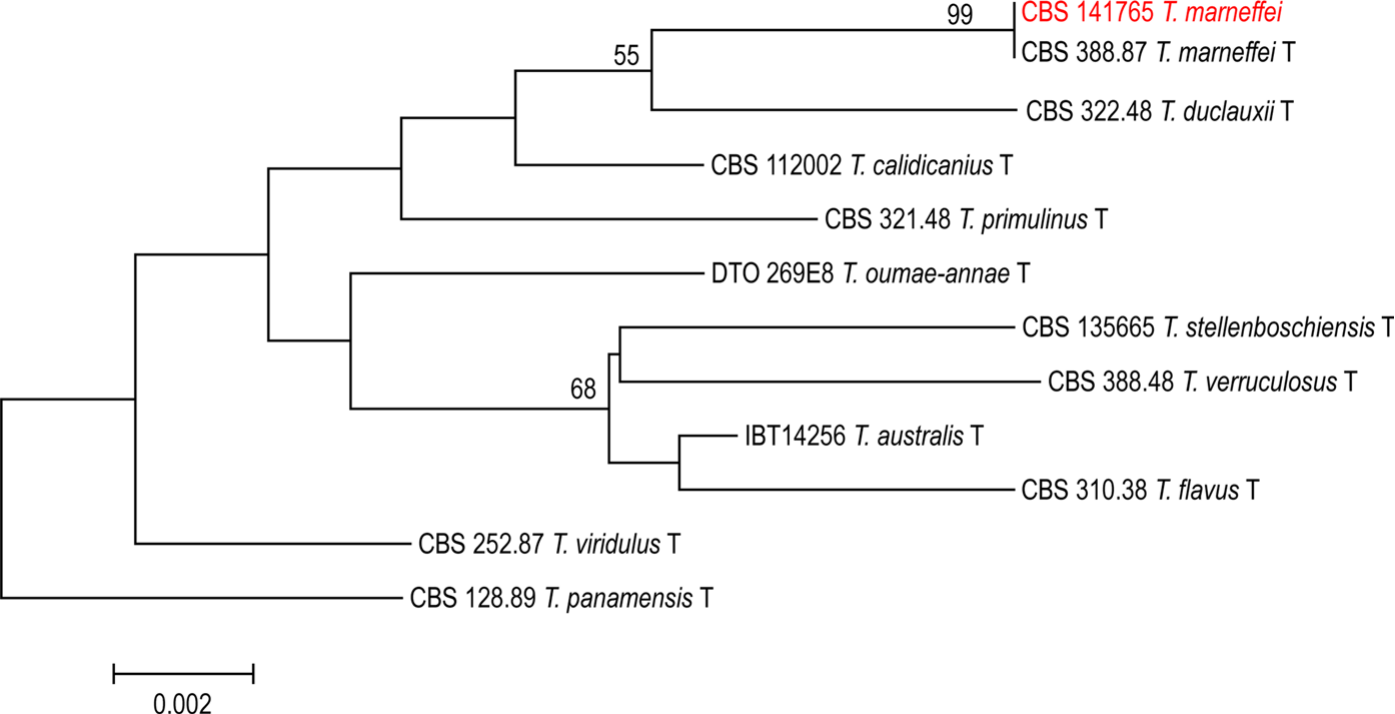
Phylogenetic tree of *Talaromyces* section (representative of type strains of closely related species to *T. marneffei*) inferred from ITS based on maximum likelihood analysis. Bootstrap values above 50% are indicated at the nodes. Our strain is indicated with *red* color. (Color figure online)

## Laboratory Investigations

Laboratory results revealed low hemoglobin (9.6 g/dl), thrombocytopenia (52 × 10^9^/L), leukocytopenia (1.1 × 10^9^/L) with neutrophilia (0.7 × 10^9^/L) and lymphocytopenia (0.3 × 10^9^/L). Blood film showed pancytopenia and low CD4 count of 100 cells/μL and an elevated C-reactive protein of 66 mg/L. Serological tests for hepatitis-B and hepatitis-C viruses and syphilis were negative. HIV-1 was confirmed by immunoblot determining the viral load as 1,754,540 copies/mL (log_10_ of 6.24). Serum galactomannan was positive, but beta-d-glucan was not done. The patient was started initially on fluconazole 400 mg once daily on day 4 of admission (21/6/16) for oral candidiasis. ATRIPLA (Efavirinz 600 mg, Emtricitiabine 200 mg and Tenofovir 300 mg) and *Pneumocystis jirovecii* prophylaxis (960 mg co-trimoxazole thrice a week) was started on day 17 of admission (3/7/16). No immune reconstitution syndrome was observed.

Blood cultures became positive after 72 h of incubation. The initial Gram stain was negative, while aerobic subcultures were positive on blood, chocolate blood agar, MacConkey agar and Sabouraud’s glucose agar after 48 h at 37 °C. Gram stain showed hyaline, septate hyphae that had the appearance of arthroconidia (Fig. 1g). All microbiological processing of the isolate was done under biosafety level 3 (BSL-3) containment. Examination of Sabouraud’s plates incubated at 25 °C after 24 h showed light green, powdery fungal colonies with a red diffusible pigment and a red colony reverse (Fig. 1e, f). Lactophenol cotton blue staining for microscopy revealed fruiting structures and spherical conidia suggestive of *Penicillium/Talaromyces* species (Fig. 1h, i). Subsequent blood cultures, to verify its significance, taken on day 6 of admission, grew the same pathogen after 72 h of incubation. Re-examination of the SGA plates at 37 °C after 72 h of incubation showed wrinkled, convoluted pink to red colonies with a non-diffusible red pigment (Fig. 1c, d). The patient also underwent a bone marrow aspiration (BMA), done on day 9 of admission in view of pancytopenia. Further staining with Periodic acid-Schiff (PAS) and GMC revealed no fungal elements. *Mycobacterium tuberculosis* cultures remained negative. A BMA sample was also sent for microbiological culturing and showed similar fungal growth as in the blood culture. There were no sputum or BAL specimens submitted for cytology or routine bacteriology and mycology cultures.

In view of the above microbiological findings, symptoms and a history of travel to Southeast Asia, the possibility of *Talaromyces* (*Penicillium*) *marneffei* infection was suspected. Therapy was initiated with liposomal amphotericin B 200 mg on day 9 of admission (26/6/16) that was continued for 1 week then shifted to oral voriconazole 200 mg twice daily after a loading dose that was given for a total of 2 weeks. He started also, as per the neurology team advice, on low-dose amitriptyline (20 mg at night) plus gabapentin (1000 mg per day) to control his neuropathic pain. The inflammatory markers decreased during treatment: C-reactive protein: (17/6: 66), (22/6: 26), (2/7: 11) and (12/7: 3). Neutrophils: (17/6: 0.7), (20/6: 0.8), (27/6: 2.9) and (2/7: 3.3). The patient was discharged on 4/7/16. One week after discharge, he was seen at the outpatient clinic, and repeated blood culture was subsequently negative. There was an excellent response to the therapy.

Further identification of the fungus was undertaken at the Westerdijk Fungal Biodiversity Institute in Utrecht, The Netherlands, under accession number CBS 141765. Sequencing of the rDNA internal transcribed spacer region (ITS) and the partial β-tubulin (*BT2*) gene was performed. Blast results with sequences in GenBank with ITS revealed 100% identity with *T. marneffei* (accession No. AB353910.1) and with β-tubulin 100% identity with *T. marneffei* (accession No. JX091389.1). Sequences of this human pathogen (CBS 141765) were deposited in GenBank with accession numbers KY115196 for ITS and KY115197 for *BT2*, respectively. Antifungal susceptibility testing performed with broth microdilution according to CLSI M38A resulted in the following MICs: amphotericin B, 0.25 mg/L; fluconazole, 4 mg/L; posaconazole, <0.016 mg/L; itraconazole, 0.031 mg/L; voriconazole and isavuconazole, 0.016 mg/l; and both anidulafungin and micafungin, 0.031 mg/L.

## Discussion

Talaromycosis is a disseminated infection caused by the fungus *Talaromyces* (*Penicillium*) *marneffei*. The disease has been reported in immunocompromised but occasionally also in otherwise healthy hosts [12]. Infection with *T. marneffei* is an endemic disease in patients with T cell immunodeficiency in Southeast Asia especially Northern Thailand, Vietnam and southern China, but extending to Taiwan, Singapore, Malaysia, Indonesia and India [13]. Locally acquired infections outside these endemic areas are extremely rare. One case was reported from West Africa in an HIV-positive patient originating from Ghana who had never travelled to tropical Asia [14]. Cases among Africans who have travelled to endemic countries [8] are much more common. Talaromycosis in HIV-positive individuals may occur when CD4 counts fall below 100 cells/μL. Most common symptoms of talaromycosis are fever, weight loss, lymphadenopathy, nonproductive cough, hepatosplenomegaly and anemia. Many patients present with multiple papular skin lesions in the face, neck, trunk and upper limbs. About one third of patients have a cough and pulmonary symptoms [15]. The mode of infection of *T. marneffei* is unclear, as the only established hosts are bamboo rats (*Rhizomys* and *Cannomys* spp.) and humans [16], and host-to-host transmission is not known to occur.

Determination of whether a patient has visited an area of endemicity is essential for rapid diagnosis of *T. marneffei*. The present case describes clinical and mycological findings of the first imported case of *Talaromyces marneffei* to Oman and the Middle East. The patient was initially clinically diagnosed as having a Varicella Zoster Virus (VZV) infection, which was, however, not verified. The patient had HIV infection with very low CD4 counts and typical skin lesions suggestive of *T. marneffei*.

Diagnosis of talaromycosis is usually achieved by staining, culture, serologic and molecular methods. Final confirmation is obtained by histopathology of intracellular arthroconidia and culture. *Talaromyces marneffei* can be observed in histopathological sections stained with hematoxylin and eosin, Grocott methenamine silver, or periodic acid-Schiff stain [12]. The organism also can be identified on peripheral blood smear or bone marrow aspirate [17]. Blood cultures are frequently positive, while bone marrow cultures are positive in nearly all cases [17].

The fungus grows as a mold at room temperature and converts to a ‘yeast-like’ (arthroconidial) form when incubated at 37 °C. This dimorphism is not found in any other member of the genus *Talaromyces*, which contains numerous penicillium-like species with colonies that exude red pigments into the agar [18, 19]. The fungus was initially misidentified as *Geotrichum* or *Trichosporon* because of the arthroconidia in the Gram stain of slides taken at 37 °C. Final diagnosis of the case as disseminated *T. marneffei* infection was made by presence of the arthroconidia, growth of the fungus from BMA and blood and by molecular identification of the pathogen as *Talaromyces* (*Penicillium*) *marneffei*.

The clinical outcome of talaromycosis can be fatal if it remains undiagnosed and untreated. Among HIV-infected patients with low CD4 counts, talaromycosis is an AIDS-defining diagnosis [20]. Additional cutaneous manifestations of translucent papular lesions, umbilicated with a central necrotic depression at the face and extremities characterizing talaromycosis [21], were also presented in our patient. Such lesions are similar to those observed in other fungal infections, particularly cryptococcosis and histoplasmosis, and were recently also reported from *Emergomyces africanus* infections [22, 23]. However, the occurrence of these skin lesions in the context of fever, cough, dyspnea, weight lost, fatigue and the presence of arthroconidia, with the evidence of patient’s travel history to an endemic area, suggested infection by *T. marneffei.*

The infection is limited to Southeast Asia. Exceptional autochthonous *T. marneffei* infections have been reported in other parts of the world such as Ghana and Togo in Africa [14, 21]. In China, prevalence of *T. marneffei* infections ranged from 1.4 to 9.4%, with reports from Beijing, 1.4% from 2009 to 2012 in HIV infection, Wuhan, Hubei 4.8% of AIDS-related hospital admission from (2006 to 2013) and 9.4% of HIV-positive patients in Guangzhou from (2004 to 2011) [24]. Patients with AIDS and talaromycosis present all over the world and mostly following travel history. Hence, a detailed travel history is important in the diagnostic workup of immunocompromised patients.

In vitro susceptibility testing showed that the fungus had low MICs of amphotericin B, posaconazole, itraconazole, voriconazole, isavuconazole, anidulafungin, and micafungin. Fluconazole was the least active. The CDC recommends 2 weeks of intravenous liposomal amphotericin B (3–5 mg/kg body weight) for *T. marneffei* infections and then oral itraconazole (400 mg qd) for 10 weeks as a standard treatment for an HIV-infected patient [25]. In conclusion, we report a case of *T. marneffei* infection in a HIV-positive patient presumably infected in Southeast Asia. It is important to consider an infection with *T. marneffei* in HIV-infected patients when international travel to endemic regions is involved.
